# B7-H4 expression in bladder urothelial carcinoma and immune escape mechanisms

**DOI:** 10.3892/ol.2014.2522

**Published:** 2014-09-11

**Authors:** WEI-HUI LIU, YA-YU CHEN, SHAO-XING ZHU, YI-NING LI, YI-PENG XU, XUE-JING WU, YI-HONG GUO, JIA-LIANG WANG

**Affiliations:** 1 Department of Urinary Surgery, Second Affiliated Hospital of Fujian Medical University, Quanzhou, Fujian 362000, P.R. China; 2 Department of Stomatology, Second Affiliated Hospital of Fujian Medical University, Quanzhou, Fujian 362000, P.R. China; 3 Department of Urinary Surgery, Zhejiang Cancer Hospital, Hangzhou, Zhejiang 310022, P.R. China; 4 Department of Pathology, Fujian Medical University Union Hospital, Fuzhou, Fujian 350001, P.R. China

**Keywords:** B7-H4, soluble B7-H4, immunohistochemistry, ELISA, bladder urothelial carcinoma, tumor immune escape

## Abstract

B7-H4 is a recently identified member of the B7 family considered to negatively regulate the immune response, and has been associated with the occurrence and development of certain types of tumor. However, little is known regarding the importance of human B7-H4 expression in bladder urothelial carcinoma. In the present study, B7-H4 expression in the tissues and sera of patients with bladder urothelial carcinoma was investigated, along with the clinical significance. In addition, the effects of activated T-lymphocyte *in vitro* cytotoxicity in the BIU-87 bladder cancer cell line following the blockade of the B7-H4 signaling pathway were also analyzed. The results showed that in normal bladder tissues, B7-H4 was not detected, but in the bladder urothelial carcinoma tissue samples, B7-H4 was detected in 24/49 (49.0%) specimens. Additionally, positive B7-H4 expression was significantly associated with increased TNM stage and pathological grade (P<0.05). Compared with the healthy control group, the serum-B7-H4 (sB7-H4) concentrations in the patients were also significantly increased (P<0.05). The sB7-H4 concentrations in cases with high-grade histology were significantly higher than those in patients with low-grade histology (P<0.05). Following the blockade of the B7-H4 antigen in BIU-87 cells, the cytotoxic activity of activated T cells against such BIU-87 cells was significantly enhanced compared with that against the control BIU-87 cells. This occurred in a T cell density-dependent and blocking antibody dose-dependent manner. These observations suggest that B7-H4 is involved in tumor occurrence, and the development and immune escape of bladder urothelial carcinoma cells. Therefore, B7-H4 may be an important target in the diagnosis and/or treatment of bladder urothelial carcinoma.

## Introduction

Bladder cancer (BCa) is one of the most common types of malignant tumor of the urinary system, worldwide. In Western countries, BCa is the fifth most common type of cancer and is a chronic disease with varying oncological outcomes. Patients require frequent follow-up and repeated treatment, rendering the cost per patient between diagnosis and mortality the highest of all types of cancer (1). In China, the annual incidence of BCa in 2002 was ~3.8/10 million in males and ~1.4/10 million in females (2). The most commonly diagnosed histological type of BCa is urothelial carcinoma (or transitional cell carcinoma), which accounts for ~95% cases. In urothelial carcinoma, >70% cases are classified as non-muscle-invasive bladder cancer (NMIBC). Remission is achieved in the majority of NMIBC cases by transurethral resection of the bladder tumor; however, after 3–5 years, the recurrence rate reaches 60–90%. Postoperative intravesical chemotherapy and Bacillus Calmette-Guerin (BCG) treatment may reduce the recurrence and progression of the disease, but the optimal infusion doses and treatment times have not been standardized. In addition, certain patients suffer recurrence and progression following treatment (1). Muscle-invasive BCa cases require partial or total bladder resection. A number of clinicians have expressed concern regarding how to inhibit the recurrence and progression of BCa.

Recent studies indicate an association between the occurrence and development of malignant tumors and immune escape. Tumor immunological studies have suggested that the T-cell-dependent immune response is the primary cellular antitumor immune response (3–5). The activation of T cells requires two signals. The first signal, termed the specific antigen stimulation signal, is generated when the antigenic peptide-major histocompatibility complex (MHC) on the antigen-presenting cells (APCs) binds to the T-cell receptor (TCR)-CD3 complex on T cells. The interaction between B7 family molecules on the APC and CD28 family molecules on the T cells provides the second signal, which is known as the costimulation signal (3). The presence of coinhibitory molecules, abnormally expressed in tumor cells, is one of the most important immune escape mechanisms (6). B7-H4 is a recently identified member of the B7 family considered to be predominantly expressed in full-time APCs, freshly isolated T cells, B cells and monocytes. Subsequent to combining with the corresponding receptor, B7-H4 is involved in tumor immune escape by suppressing specific cellular and humoral immunity, and inducing specific T-cell apoptosis (7–9). In several types of tumor tissue, including those of ovarian cancer (10), lung cancer (11), renal cell carcinoma (12), breast cancer (13) and prostate cancer (14), high levels of B7-H4 protein expression have been identified and found to be closely associated with tumor development, invasion and metastasis. Studies have shown that tumor cells directly bind T-cell surface receptors through expressing B7-H4 protein or secreting soluble B7-H4 (sB7-H4) to inhibit the proliferation of CD4^+^ T cells, block the T-cell division cycle, and inhibit the release of antitumor cytokines and CD8^+^ T-cell cytotoxic activity against tumor cells (15,16). However, whether B7-H4 is expressed in BCa remains unclear, as, to the best of our knowledge, no studies investigating this association have been reported.

The prognosis of patients with BCa treated with infusion therapy using BCG or combined cytokines, such as interleukin 2 (IL-2), has been shown to be superior to that of patients with tumors treated with chemotherapeutic drugs. Additionally, BCG remains the most effective treatment for the prevention of moderate- and high-risk NMIBC recurrence and progression following surgery (17–21). Therefore, tumor immune escape may be involved in the incidence and development, or recurrence and progression of BCa. The present study focused on B7-H4 expression in bladder urothelial carcinoma (part one) and investigated the association between B7-H4 and immune escape mechanisms in this type of cancer (part two).

## Materials and methods

### Clinical data

Bladder urothelial carcinoma tissue specimens were obtained by surgical resection or partial removal of the bladder from 49 patients (42 males and 7 females; age range, 44–84 years; 30 newly diagnosed cases and 19 recurrent cases), between January 2009 and August 2011 at Fujian Medical University Union Hospital (Fuzhou, China). These specimens were divided into 32 cases of non-muscle-invasive tumor (T_a_–T_1_) and 17 cases of muscle-invasive tumor (T_2_–T_4_) by the 2009 Union for International Cancer Control standards (22). Histological grading, conducted according to the 2004 World Health Organization classification criteria (23), divided the specimens into 15 low-grade cases and 34 high-grade cases.

Urothelial cancer serum samples were selected from 45 clinically diagnosed cases from Fujian Medical University Union Hospital, which were pathologically confirmed following surgery (33 males and 12 females; age range, 35–87 years; 36 initial onset cases and 9 recurrent cases). The TNM staging standards and histological grading criteria were defined as above; 32 non-muscle invasive (T_a_–T_1_) cases and 13 muscle invasive (T_2_–T_4_ stage) cases, and 34 low-grade cases and 11 high-grade cases were identified. In the control group, 45 healthy individuals with normal physical examination and no identified tumors, 26 males and 19 females, aged 31 to 78 years, were selected at Fujian Medical University Union Hospital. All patients were confirmed as negative for autoimmune diseases and other types of tumor, and had not undergone any preoperative therapy. This study was approved by the ethics committee of Fujian Medical University Union Hospital (Fuzhou, China). Written informed consent was obtained from all patients.

### Cell maintenance

BIU-87 human bladder urothelial carcinoma cells was purchased from Wuhan Cell Bank (Wuhan, China) and maintained in RPMI 1640 medium (Fuzhou DingGuo Biotechnology Co., Ltd., Fuzhou, China) supplemented with 10% fetal bovine serum at 37°C in an atmosphere of 5% CO_2_. T lymphocytes isolated from the peripheral blood of a healthy 45 year-old male were maintained in RPMI-1640 medium supplemented with 10% fetal calf serum (Gibco-BRL, Carlsbad, CA, USA).

### Part one

#### Immunohistochemistry

Immunostaining was performed on the BCa samples using the Polink-2 Plus^®^ horseradish peroxidase (HRP) Polymer Detection system for monoclonal rabbit anti-human primary antibody (PV-9001; GBI Labs, Mukilteo, WA, USA). Resected tissue specimens were fixed in formalin, embedded in paraffin and cut into 5-μm serial sections. The slides were deparaffinized with xylene and dehydrated in graded alcohol. Subsequent to retrieval of the antigen by heating in a microwave oven for 1–2 min, the slides were incubated with 0.3% H_2_O_2_ solution in methanol for 20 min to block endogenous peroxidase activity. Following three washes with phosphate-buffered saline (PBS), the slides were incubated in 1.5% goat serum to block nonspecific background staining. The sections were incubated with polyclonal rabbit anti-B7-H4 (diluted 1:200; R&D Systems, Minneapolis, MN, USA) in a humid chamber at 4°C overnight. Subsequent to washing with PBS, the sections were incubated successively with polymer Helper and poly-HRP goat anti-rabbit IgG (Beijing Zhongshan Golden Bridge Biotechnology Co., Ltd., Beijing, China) at 37°C for 10–20 min, and were then stained with 3,3′-diaminobenzidine solution. The controls were incubated with PBS.

#### B7-H4 expression and immunohistochemical analysis

B7-H4 expression was defined as the percentage of the tumor cytoplasm or membrane with brown or tan particles. Five horizons were randomly observed at a high magnification using an optical microscope (×400; Ti-100, Nikon Corporation, Tokyo, Japan). The specimens were classified into two groups, as determined by staining intensity: Negative (<10% positive cells) and positive (10–100% positive cells). The associations between B7-H4 expression and clinical pathological parameters were analyzed using the χ^2^ and Fisher’s exact tests.

#### ELISA

For serum sample collection and preservation, the bladder urothelial carcinoma patient group and the healthy group were physically examined in the morning, and 3–5 ml peripheral fasting venous blood was siphoned, stood for 1 h and centrifuged for 3–5 min at 300 × g. The upper serum was separated carefully into a 1.5 ml Eppendorf tube using a micropipette, and cryopreserved at −20°C.

The serum samples were removed from the −20°C freezer (after <6 months) and placed in a 4°C refrigerator for 2 h. A human B7-H4 ELISA kit (Shanghai, BlueGene Biotech Co., Ltd., Shanghai, China) was removed from a 4°C refrigerator and maintained at 20–25°C for 15–30 min. Following the manufacturer’s instructions, 50 μl standard solution, 50 μl sample or 50 μl distilled water serving as a blank control was added to each blank micropore of the microtiter plates. Subsequently, 100 μl enzyme-labeled solution was added to each well, excluding the blank control wells. The microtiter plates were incubated for 1 h after sealing with sealing compound at 37°C in the incubator to maintain a stable temperature and humidity. The plates were fully cleaned five times and adequate pressure was maintained in each well using concentrated detergent solution diluted 1:100 in distilled water. The plates were thoroughly washed and patted dry with absorbent paper. A volume of 50 μl chromogenic agent A,B solution was added to each well. Following 15 min reaction time in the dark at 20–25°C, 50 μl stop solution was added to each well to terminate the reaction. Each sample was assayed in triplicate.

#### Calculation of sB7-H4 concentration and statistical analysis

The optical density (OD) for each well was measured using a microplate reader (Epoch, BioTek Instruments, Inc., Winooski, VT, USA) under a 450-nm wavelength for 30 min. A curve was drawn as determined by the standard OD values, with ordinate and standard concentrations as abscissae, and the concentration of each of the samples was calculated according to the standard curve (sensitivity, 0.1 ng/ml). The associations between sB7-H4 concentration and clinical pathological parameters were analyzed using Student’s t-test. SPSS version 13.0 (SPSS, Inc., Chicago, IL, USA) and Microsot Excel 2007 (Microsoft Corporation, Redmond, WA, USA) were used for all statsitical analyses. P<0.05 was considered to indicate a statistically significant difference.

### Part two

#### Immunocytochemistry

The BIU-87 BCa cells were adhered on coverslips and fixed with 4% paraformaldehyde. The coverslips were incubated in 1.5% goat serum to block nonspecific background staining. The remaining steps performed and the antibodies used were the same as for the tissue staining, as described above.

#### Isolation of peripheral blood mononuclear cells (PBMCs) by density gradient centrifugation

A volume of 4 ml healthy human peripheral blood was injected into sterile vials containing heparin, and the solution was mixed thoroughly subsequent to the addition of an equal volume of Hanks’ solution. Subsequently, 8 ml of this mixture was slowly injected along the wall of a centrifuge tube containing 4 ml lymphocyte separation medium, in order to overlap in the separation medium (ratio 2:1). The tube was centrifuged at 200 × g for 15–20 min. The buffy coat cells were pipetted off carefully using a capillary pipette.

#### Separation of lymphocytes by adherent separation

The PBMCs, including monocytes and lymphocytes, were seeded in a culture flask, which was well-agitated, and placed in an incubator thermostat (37°C and 5% CO_2_) for 2 h, then the cell suspension was carefully pipetted off.

#### Transformation of T lymphocytes

Designated lymphocyte densities (1×10^6^–4×10^6^/ml), were added to different concentrations of concanavalin A (ConA; 1, 2, 4 and 8 μg/ml) for 48 h, then IL-2 (100 IU/ml; Jiangsu Kingsley Pharmaceutical Co., Ltd., Jiangsu, China) was added to maintain activation. The T-lymphocyte appreciation rate was detected using Cell Counting Kit-8 (CCK-8; Dojindo, Kunamoto, Japan).

#### Proliferation rate calculation

The different lymphocyte densities stimulated with the various concentrations of ConA were inoculated in 96-well plates. OD_450nm_ values were measured using the microplate reader subsequent to the addition of 10 μl CCK-8 reagent to each hole. The stimulation index (SI) was calculated using the following equation: SI = (ConA group OD_450nm_ value − medium group OD_450nm_ value)/(unstimulated group OD_450nm_value − medium group OD_450nm_ value). Each condition was established in three wells. The cell density with the average highest proliferation rate was selected for subsequent experiments.

#### Identification of T lymphocytes by the E-rosette test

A volume of 1 ml T lymphocytes (density 1×10^6^/ml) was centrifuged at 200 × g for 10 min, and then the cell supernatant was discarded. Equal quantities of 10% RPMI-1640 liquid were added to the T lymphocytes to resuspend the cells. Subsequently, 0.2 ml 1% sheep red blood cells (SRBCs; Fuzhou DingGuo Biotechnology Co., Ltd.) were mixed with the T-cell suspension and the solution was maintained at 4°C overnight. On the following day, 0.1 ml 0.8% glutaraldehyde was added dropwise to the mixture of cells. Subsequent to fixing the cells with formalin, Wright’s staining method was used for cell staining.

#### Cytotoxicity assay

BIU-87 cells in the logarithmic growth phase were divided into experimental and control groups. Mouse anti-human B7-H4 monoclonal antibodies (mAbs) for sterile environments (R&D Systems) were added to the cells in the experimental group at different concentrations (0, 5, 10, 15 and 20 μg/ml), and equal quantities of monoclonal mouse anti-human IgG2b antibody (eBioscience, Inc., San Diego, CA, USA) were added to the controls. The two groups were cultured in an incubator at 37°C with 5% CO_2_ for 1 h.

T lymphocytes stimulated by ConA and IL-2 were designated effector cells, and BIU-87 cells were termed target cells. The two groups of cells (100 μl each) were mixed together in 96-well plates according to different density ratios (effector to target cells, 10:1, 20:1 and 30:1) in an incubator for 48 h at 37°C and 5% CO_2_ (Table I). The mixed cell solution was termed the experimental group. Equal numbers of effector cell and target cell wells were simultaneously independently set. Each experimental condition was repeatedly analyzed in three wells. The OD_450nm_ values were measured using the microplate reader subsequent to the addition of 10 μl CCK-8 reagent to each well. Cytotoxic activity was calculated using the following formula: [1−(experimental well OD_450nm_ value − effector cell OD_450nm_ value)/target cell OD_450nm_ value] × 100%.

#### Statistical analysis of cytotoxicity assay

The results are expressed as the mean ± standard deviation and the groups were compared using Student’s t-test. SPSS version 13.0 (SPSS, Inc.) and Microsoft Excel 2007 (Microsoft Corporation) were used for all statistical analyses. P<0.05 was considered to indicate a statistically significant difference.

## Results

### Part one

#### Immunochemistry

**Figure 1:**
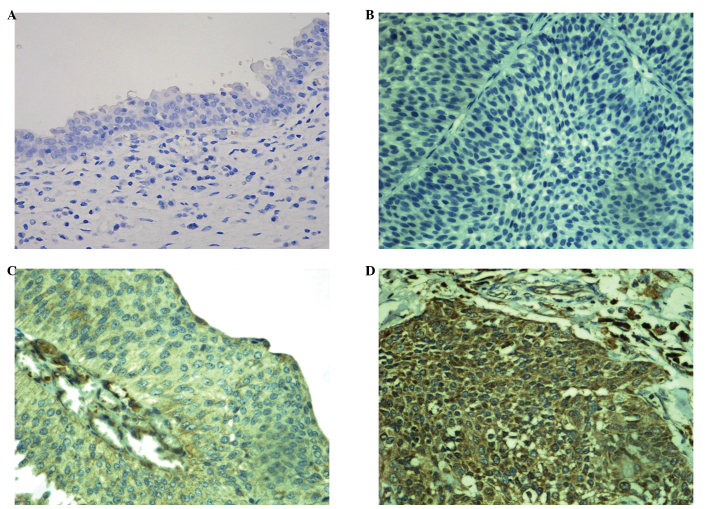
B7-H4 expression in normal bladder tissues and urothelial carcinoma tissues (3,3′-diaminobenzidene staining; magnification, ×400). (A) No expression in normal bladder tissue. (B) Negative expression in bladder urothelial carcinoma. (C) Positive expression in low-grade urothelial carcinoma. (D) Positive expression in high-grade urothelial carcinoma.

B7-H4 was not detected in the normal bladder tissue samples. In the bladder urothelial carcinoma samples, the positive rate of B7-H4 expression was 49.0% (24/49). Both weak (low-grade) and strong (high-grade) immunostaining intensity was demonstrated (Fig. 1). B7-H4 expression was clearly associated with clinical stage and pathological grade, as the positive rate of B7-H4 expression in patients at stage T_2_–T_4_ was significantly higher than that in patients at stage T_a_–T_1_ (P<0.05). Furthermore, the positive rate of B7-H4 expression in high-grade cases was significantly higher than that in low-grade cases (P<0.05). Although the positive expression rate in the infiltration group was higher than that in the non-infiltration group, the difference was not statistically significant (P>0.05). No significant differences (P>0.05) were detected between B7-H4 expression and the other pathological parameters (Table II).

#### ELISA

**Figure 2:**
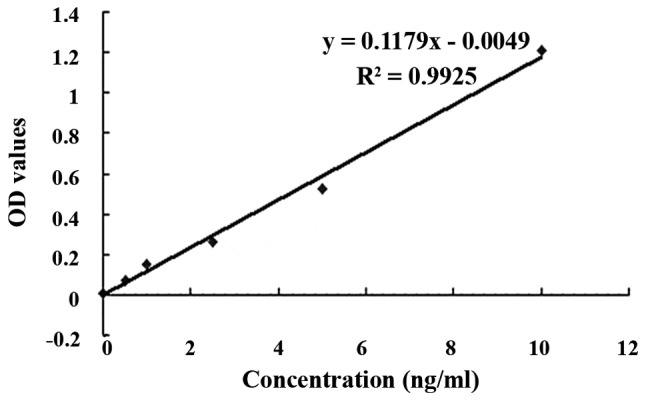
Standard curve of soluble B7-H4 concentrations. OD, optical density.

A standard curve was generated with standard B7-H4 concentrations (0, 0.5, 1.0, 2.5, 5.0 and 10.0 ng/ml) as the abscissae and the corresponding OD values as the ordinates. This was used to produce an equation: Y = 0.1179X − 0.0049, R^2^=0.9925, to determine the sB7-H4 concentrations in serum from the case and control patients (Fig. 2). The sB7-H4 concentrations in the case group were significantly increased compared with those of the healthy control group (P<0.05; Table III). Analysis of the differences between groups with different urothelial carcinoma histological grades revealed that patients with high-grade histology exhibited significantly higher sB7-H4 concentrations than patients with low-grade histology (P<0.05; Table IV). However, no statistically significant differences in sB7-H4 concentration were detected between groups classified by other factors.

### Part two

#### Transformation of T lymphocytes

**Figure 3:**
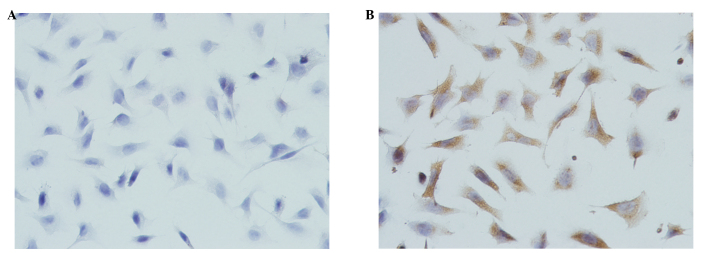
B7-H4 expression in BIU-87 human bladder urothelial carcinoma cells (3,3′-diaminobenzidene staining; magnification, ×400). (A) Unstained in the control group. (B) Positive expression in BIU-87 cells.

Immunocytochemical analysis revealed positive B7-H4 expression in the BIU-87 cells incubated with B7-H4 mAbs, and negative expression in the control BIU-87 cells incubated with PBS instead of primary antibody (Fig. 3). The SI results revealed that optimal lymphocyte proliferative activity occurred when the cell density was set at 2×10^6^ cells/ml and the ConA concentration was set at 4 μg/ml (P<0.05) when compared with the other ConA concentrations (1, 2 and 8 μg/ml). Thus, lymphocytes at a density of 2×10^6^ cells/ml were used as effector cells.

#### E-rosette test

**Figure 4:**
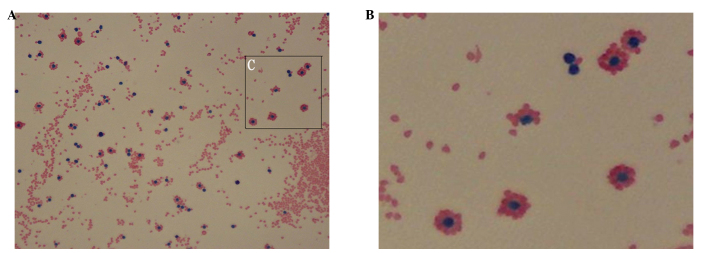
T lymphocytes (stained blue) surrounded by sheep red blood cells (stained red), forming rosettes (stained by Wright’s method), at a (A) magnification, ×400 and (B) magnification, ×800 (amplified from C).

For the identification of T lymphocytes, Wright’s staining method was used. SRBCs were stained red and lymphocytes were stained blue. ‘Rosette cells’, with one lymphocyte surrounded by ≥3 SRBCs were identified. The blue cells at the centers of these rosette cells were demonstrated to be mature T lymphocytes (Fig. 4). The calculation formula for the rosette formation rate was as follows: Rosette formation rate (%)= bound T lymphocytes (n)/[bound + unbound T lymphocytes (n)]. The results demonstrated that activated T-lymphocyte purity reached 80%, which was similar to the purity previously reported in the literature (26).

#### Cytotoxicity assay

To examine activated T-lymphocyte cytotoxicity against BIU-87 human bladder urothelial carcinoma cells following blockade of the B7-H4 signaling pathway *in vitro*, control and B7-H4 mAB-stimulated BIU-87 cells were mixed with the activated T lymphocytes at different ratios. When the mouse anti-human B7-H4 mAb concentration used to block B7-H4 activity in the BIU-87 cells was 10 μg/ml, cytotoxicity was significantly greater than that in the normal BIU-87 cells (P<0.01 or P<0.05). The cytotoxic effect was significantly enhanced and the T-lymphocyte concentration was significantly increased following blockade (P<0.01 and P<0.05; Table V).

## Discussion

In part one, the study focused on B7-H4 expression in 49 cases of human bladder urothelial carcinoma tissues and the adjacent tissues. A total of 24 cases (49.0%) were found to exhibit positive B7-H4 expression, whereas the adjacent bladder tissue did not express B7-H4. In the cancer tissue specimens, B7-H4 was expressed and located in the cytoplasm and plasma membranes of the cancer cells, a finding consistent with studies examining B7-H4 expression in non-small-cell lung cancer (11) and breast cancer tissues (13). However, in renal clear cell carcinoma, B7-H4 was shown to be expressed only on the cell membrane (12). In the present study, B7-H4 expression was shown to be closely associated with increased TNM stage and histological grade in the bladder urothelial carcinoma samples, as the positive expression rate in the myometrial invasion group was significantly higher than that of the non-muscle-invasive group, and the positive expression rate in the high-grade group was significantly higher than that in the low-grade group.

Bladder urothelial carcinoma cells may inhibit T-cell activity and induce apoptosis in tumor antigen-specific cells due to B7-H4 combining with the corresponding T-cell surface receptor. These mechanisms may cause bladder tumor immune escape to occur, and contribute to bladder urothelial carcinoma development and progression. Thus, the detection of B7-H4 protein may reflect the degree of malignancy, and the risk of recurrence and progression in bladder urothelial carcinoma. One study by the European Organization for Research and Treatment of Cancer observed that the morphological and structural diversity of the tumor, tumor size and short-term recurrence of BCa were important prognostic factors for recurrence or secondary recurrence (1). Important prognostic factors for tumor progression included tumor grade, stage and carcinoma *in situ*, and cases with a history of recurrence and/or high-grade histology were more likely to relapse and progress. In the present study, no significant differences were identified between the rates of B7-H4 positive expression in newly diagnosed and recurrent BCa groups; however, B7-H4 expression was closely associated with TNM stage and histological grade. Therefore, blocking B7-H4 protein activity in bladder tumor tissues may become an effective immunotherapeutic method to inhibit stage and grade progression in bladder urothelial carcinoma.

B7-H4 protein is also present in the blood and body fluids in a soluble form, sB7-H4. Tumor cells secrete sB7-H4, which restrains T-cell proliferation through blocking the cell cycle at the G_0_/G_1_ phase and further inhibits the T cell immune response by inducing T cell apoptosis (21,24). sB7-H4 levels detected in several genitourinary system tumors have been shown to have important clinical significance at diagnosis, and stage and prognosis assessment. Simon *et al* (10) found that sB7-H4 levels in serum and ascites samples from ovarian cancer patients were significantly higher than those in normal and benign lesions of the reproductive system. Another study (26) found that the combined detection of sB7-H4 and CA125 significantly improved the diagnostic yield for ovarian cancer. Thompson *et al* (24) found that the sB7-H4-positive rate and the average sB7-H4 expression levels in renal cell cancer (RCC) patients were significantly higher than those in normal individuals, and were associated with the development of lymph node and distant metastases. The authors hypothesized that sB7-H4 may become a novel serum marker in RCC diagnosis, and that sB7-H4 detection has significant value in the prediction of tumor stage and prognosis. The present study found that sB7-H4 concentrations in patients with bladder urothelial carcinoma were significantly higher than those of normal individuals. The sB7-H4 concentrations in cases with high-grade histology were significantly higher than those in the low-grade cases, but no significant differences in expression rates were identified between gender, age, TNM stage, tumor size or the initial issuance/relapse status groups. These results suggest that sB7-H4 may be involved in the development of bladder urothelial carcinoma, and that the detection of serum sB7-H4 concentrations may provide certain value in the diagnosis and pathological grade assessment of bladder urothelial cancer.

Host T lymphocytes are activated and proliferate under the effects of polyclonal activators, anti-CD3 antibody and antigen-MHC peptide complexes. T lymphocytes are involved in the host adaptive immune response only when differentiated into functional T cells (27,28). High initial T cell clonal expansion may produce a large number of effective CD4^+^ and CD8^+^ T cells, which is conducive to the rapid elimination of pathogens and the formation of memory T cells. ConA is a common polyclonal T cell activator, which activates T lymphocyte proliferation by acting on the TCR-CD3 complex on the T cell membrane (29). PBMCs include T cells, B cells and monocytes. However, the largest proportion of T cells are in PBMC, monocytes may proliferate and differentiate into macrophages *in vitro* without stimulation. Due to the adherent growth of monocytes and the suspended growth of lymphocytes, in the present study, the adherent separation method was used to remove mononuclear cells, to reduce background interference and increase the reliability of the CCK-8 analysis.

B7-H4 expression in the bladder urothelial carcinoma samples was demonstrated in part one of the present study. In order to further analyze the association between B7-H4 and bladder urothelial carcinoma immune escape mechanisms, cellular level experiments *in vitro* were conducted. The results revealed B7-H4 expression in BIU-87 BCa cells. High-purity activated T lymphocytes were obtained; following B7-H4 antigen blockade in the BIU-87 cells, the cytotoxic activity of these activated T cells against BIU-87 cells was significantly enhanced compared with that against normal BIU-87 cells, in a T-cell density-dependent and blocking antibody dose-dependent manner. The results indicate that the cytotoxic activity of the activated T cells against the BIU-87 cells was significantly enhanced following B7-H4 blockade. The B7-H4-mediated immune escape effect in BCa was reversed when this tumor antigen binding the T cell surface corresponding receptor was blocked. However, Miyatake *et al* (25) hypothesized that B7-H4 tumor cells mediate immune escape through inhibiting T-cell chemotaxis to the tumor tissue, as B7-H4 expression intensity in breast cancer was found to be inversely proportional to the number of infiltrating CD3^+^ and CD8^+^ T cells. Therefore, altering B7-H4 protein expression in bladder urothelial carcinoma cells may enhance T-cell cytotoxicity to the cancer cells, and also promote and maintain a functional T cell immune response; thus the rate of BCa recurrence and progression may be reduced.

In conclusion, the results of the present study revealed that B7-H4 was upregulated in bladder urothelial carcinoma tissues and serum samples from patients, and was closely associated with TNM stage and histological grade. Therefore, B7-H4 may be involved in the occurrence and development of BCa, and may be important in bladder urothelial carcinoma immune escape. B7-H4 expression in tissue and serum was analyzed, along with the clinical significance. Therefore, simulations of the body environment and the construction of relevant animal models may be required in further studies.

## Figures and Tables

**Table I:** Density ratio of effector cells to target cells.

T cell density (cells/ml)	BIU-87 cell density (cells/ml)
3×10^5^	1×10^4^
2×10^5^	1×10^4^
1×10^5^	1×10^4^

Quantitative cell density was detected using the Cell Counting Kit 8.

**Table II:** Associations between B7-H4 expression and clinicopathological factors.

Clinicopathological factors	Positive, n=24	Negative, n=25	Positive rate (%)	χ^2^	P-value
Gender					
Male	21	21	50.0	0.123	0.726
Female	3	4	42.9		
Age, years					
<60	9	8	52.9	0.163	0.686
≥60	15	17	46.9		
TNM stage					
T_0_–T_1_	11	21	34.4	7.873	0.005
T_2_–T_4_	13	4	76.5		
Histological grade^a^					
Low-grade	3	12	20.0	7.265	0.007
High-grade	21	13	61.8		
Tumor status					
Initial group	14	16	46.7	0.166	0.684
Recurrent group	10	9	52.6		
Infiltration degree					
Non-infiltration (T_a_)	5	11	31.3	2.988	0.084
Infiltration	19	14	57.6		

a According to the World Health Organisation grading system (23).

**Table III:** sB7-H4 concentrations (ng/ml) in the case and control groups.

Group	n	Mean ± SD
Control	45	1.664±1.316
Case	45	2.561±1.965^a^

a P<0.05, compared with the control group.

sB7-H4, serum B7-H4.

**Table IV:** sB7-H4 concentrations (ng/ml) in low- and high-grade bladder urothelial carcioma groups.

Histological grade	n	Mean ± SD
Low	34	2.178±1.881
High	11	3.745±1.811^a^

a P<0.05, compared with the low-grade group.

sB7-H4, serum B7-H4.

**Table V:** Effects of activated T-lymphocyte cytotoxicity against BIU-87 human bladder urothelial carcinoma cells following blockade of B7-H4 activity *in vitro* (%, mean ± SD).

	Density ratio (effector:target cells)		
	
Group	30:1	20:1	10:1
Control	0.6621±0.0653	0.5402±0.0557	0.3341±0.0385
Blocked	0.7987±0.0717^a^,^c^	0.6672±0.0454^b^,^d^	0.4292±0.0634^b^

a P<0.01, vs. control group;

b P<0.05, vs. control group;

c P<0.05, vs. 20:1 blocked group;

d P<0.01vs. 10:1 blocked group.
